# On the Phase Relationship between Excitatory and Inhibitory Neurons in Oscillation

**DOI:** 10.3389/fncom.2016.00138

**Published:** 2016-12-26

**Authors:** Xiaolong Zou, Da-Hui Wang

**Affiliations:** ^1^School of Systems Science, Beijing Normal UniversityBeijing, China; ^2^National Key Laboratory of Cognitive Neuroscience and Learning, Beijing Normal UniversityBeijing, China

**Keywords:** neural network modeling, oscillations, phase shift, E-I loop, I-I loop

## Abstract

Characteristic phase shifts between discharges of pyramidal cells and interneurons in oscillation have been widely observed in experiments, and they have been suggested to play important roles in neural computation. Previous studies mainly explored two independent mechanisms to generate neural oscillation, one is based on the interaction loop between pyramidal cells and interneurons, referred to as the E-I loop, and the other is based on the interaction loop between interneurons, referred to as the I-I loop. In the present study, we consider neural networks consisting of both the E-I and I-I loops, and the network oscillation can operate under either E-I loop dominating mode or I-I loop dominating mode, depending on the network structure, and neuronal connection patterns. We found that the phase shift between pyramidal cells and interneurons displays different characteristics in different oscillation modes, and its amplitude varies with the network parameters. We expect that this study helps us to understand the structural characteristics of neural circuits underlying various oscillation behaviors observed in experiments.

## Introduction

Oscillatory responses are widely observed in neural systems. It has been an active research topic for decades to unveil the origins of these oscillations and their potential roles in computation. In neural oscillation, the spiking probability of a neuron typically exhibits a peaked distribution at a fixed phase with respect to the circle of the oscillating local field potential, which is called the phase of neuronal response. Interestingly, it was found that in several brain areas the phase of pyramidal cells leads to that of interneurons by a few ms in oscillation (Fisahn et al., 1998; Csicsvari et al., 2003; Hájos et al., 2004; Hasenstaub et al., 2005; Mann and Paulsen, 2005; Mann et al., 2005a,b; Oren et al., 2006; Gulyás et al., 2010; Vinck et al., 2013; Zemankovics et al., 2013). With respect to the period of local field potential, this corresponds to a significant phase shift of 60° *in vivo* (Csicsvari et al., 2003; Vinck et al., 2013), and 55° (Mann et al., 2005a) or 23° (Hájos et al., 2004; Oren et al., 2006) *in vitro*. It has been suggested that this phase shift plays important roles in neural computation (Buzsáki and Chrobak, 1995; Maass and Natschlager, 1997; Fries et al., 2007; Nikolic, 2007; Tiesinga et al., 2008; Quiroga and Panzeri, 2009; Vinck et al., 2010). Computational models based on the interaction loop between excitatory and inhibitory neurons were also proposed to reproduce the phase shift phenomenon (Freeman, 1968; Wilson and Cowan, 1972; Leung, 1982; Börgers and Kopell, 2005; Orbán et al., 2006; Ledoux and Brunel, 2011), but so far no detailed study has been done to unveil how exactly the network structure and neuronal dynamical properties affect the amount of phase shift (Geisler et al., 2005; Wang, 2010).

Two different mechanisms have been proposed to generate neural oscillation, one is based on the feedback inhibition loop formed by excitatory and inhibitory neurons, referred to the E-I loop hereafter, and the other is based on the mutual inhibition loop formed by interneurons, referred to as the I-I loop (Fries et al., 2007; Wang, 2010). These two mechanisms give rise to different phase relationships between neurons, since their ways of generating oscillatory responses are different. Previous studies often focused on employing one mechanism in a network to generate oscillation, but in reality, a neural network typically consists of a large number of excitatory and inhibitory neurons, whose reciprocal connections form the E-I and I-I loops simultaneously, and the interplay between these two types of loop determines the oscillatory mode of the network. Thus, in this study, we go beyond previous works by considering neural networks composed of both the E-I and I-I loops. We explore how the interplay between two loops determines the phase relationship between pyramidal cells and interneurons, and how the amount of phase shift between neurons varies with the parameters. We expect that this study helps us to understand the characteristics of neural circuits that generate various oscillations as observed in experiments.

## Materials and methods

### Network architecture

**Figure 1:**
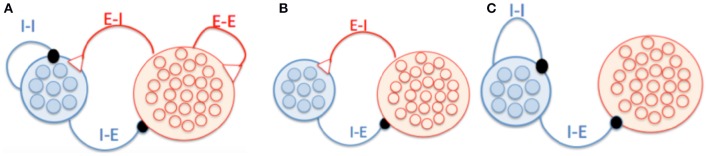
**Schematic of network architecture. (A)** A network model in which neurons form the E-I and I-I loops. **(B)** A network model having the E-I loop alone. Pyramidal cells and interneurons are reciprocally connected, and there is no connection between neurons of the same type. **(C)** A network model having the I-I loop alone. There are connections from interneurons to pyramidal cells but no feedback connections.

This study aims to explore the phase relationship between excitatory and inhibitory neurons in oscillation of neural networks. For the convenience of illustration, we organized neurons into two groups, one for pyramidal cells and the other for interneurons, to highlight how the E-I and I-I loops are formed respectively (Figure 1A), although in practice they may be mixed in the space. To elucidate the mechanism for phase shift clearly, we first considered two extreme scenarios when the network oscillation is controlled by either the E-I loop alone (Figure 1B) or the I-I loop alone (Figure 1C). In the network with the E-I loop alone, pyramidal cells, and interneurons are reciprocally connected, but no connection between neurons of the same type. In the network with the I-I loop alone, interneurons are reciprocally connected among themselves, and they also projected to pyramidal cells but no feedback connection exists. In the network with both the E-I and I-I loops, neurons of the same, or different types are also reciprocally connected with each other. The connectivity is a sparse random graph. Each pair of neurons is connected at probability of 10%. In the simulations for all the above network models, 4000 pyramidal cells, and 1000 interneurons were used.

### Dynamics of interneuron

An interneuron is modeled as single compartment fast-spiking Hodgkin-Huxley type model slightly modified from Wang and Buzsáki (1996), which is written as:

(1) $$\begin{array}{l} C_{m}\frac{dV\left(t\right)}{dt}=\;-\;I_{L}-I_{Na}-I_{K}-I_{syn}+I_{ext} \end{array}$$

where *C*_*m*_ = 1 nF and *I*_*ext*_ is external input. The leak current *I*_*L*_ = *g*_*L*_(*V*−*E*_*L*_) with *g*_*L*_ = 0.02 μS and *E*_*L*_ = −67 mV. The spike-generation currents are $I_{Na}=g_{Na}m^{3}h\left(V-E_{Na}\right)$, $I_{K}=g_{K}n^{4}\left(V-E_{K}\right)$. The variables *m, h*, and *n* follow the first-order kinetics, $\frac{dx}{dt}=\;\phi_{x}\left(a_{x}\left(1-x\right)-\beta_{x}x\right)$, where subscript *x* denotes *m, h*, or *n*. *a*_*m*_(*V*) = −0.1(*V* + 35) / (−1 + exp(−0.1(*V* + 35))), β_*m*_(*V*) = 4 exp(−(*V* + 60)/18), *a*_*h*_(*V*) = 0.07 exp(−(*V* + 58)/20), β_*h*_(*V*) = 1 / (1 + exp(−0.1(*V* + 28))), *a*_*n*_(*V*) = −0.01(*V* + 34) / (−1 + exp(−0.1(*V* + 34))), β_*n*_(*V*) = 1 / (1 + exp(−0.1(*V* + 44)/80)), *g*_*Na*_ = 14 μS, *g*_*K*_ = 1.8 μS, *E*_*Na*_ = 55 mV, *E*_*K*_ = −80 mV, ϕ_*m*_ = ϕ_*h*_ = ϕ_*n*_ = 5.

### Dynamics of pyramidal cell

A pyramidal cell is modeled as two compartments Hodgkin-Huxley type model slightly modified from Geisler et al. (2005), which is written as

(2) $$\begin{array}{r} C_{m}\frac{dV_{s}}{dt}=\;-\;I_{L}-I_{Na}-I_{K}-g_{sd}\left(V_{s}-V_{d}\right)/p-I_{syns}\\ \;+\;I_{ext} \end{array}$$

(3) $$\begin{array}{r} C_{m}\frac{dV_{d}}{dt}=\;-\;I_{L}-I_{Ca}-I_{ahp}-g_{ds}\left(V_{d}-V_{s}\right)/\left(1-p\right) \end{array}$$

where *C*_*m*_ = 0.25 nF. The spike generation currents *I*_*Na*_ and *I*_*K*_ have same dynamics as those of fast spiking interneurons except *a*_*m*_(*V*) = −0.1(*V* + 33) / (−1 + exp(−0.1(*V* + 33))), β_*m*_(*V*) = 4 exp(−(*V* + 58)/12), *a*_*h*_(*V*) = 0.07 exp(−0.1(*V* + 50)), β_*h*_(*V*) = 1 / (1 + exp(−0.1(*V* + 20))), *a*_*n*_(*V*) = −0.01(*V* + 33) / (−1 + exp(−0.1(*V* + 33))), β_*n*_(*V*) = 0.125 exp(−0.04(*V* + 44)). The conductances are set as *g*_*Na*_ = 11.25 μS, *g*_*K*_ = 4.5 μS, *g*_*L*_ = 0.025 μS. The reversal potentials are set as *E*_*Na*_ = 55 mV, *E*_*K*_ = −80 mV, *E*_*L*_ = −65 mV. The high threshold calcium current in the dendrite is $I_{Ca}=g_{Ca}m_{\infty }^{2}\left(V-E_{Ca}\right)$, where *m* is assumed fast variable and is replaced by its steady state *m*_∞_ = 1 / (1 + exp(−(*V*_*d*_ + 20)/9), *g*_*Ca*_ = 0.25 μS, *E*_*Ca*_ = 120 mV. The voltage-dependent, calcium activated potassium current: $I_{AHP}=g_{AHP}\left(V-V_{K}\right)\left[Ca^{2+}\right]/\left(\left[Ca^{2+}\right]+30\mu \text{M}\right)$. The intracellular calcium follows dynamics: $d\left[Ca^{2+}\right]/dt=-\alpha I_{Ca}-\left[Ca^{2+}\right]/\tau_{Ca}$ with α = 4 μM / (*ms μA*) and τ_*Ca*_ = 80 ms, *g*_*AHP*_ = 1.25 μS.

### Synaptic dynamics

The synaptic currents are mediated byα-amino-3-hydroxy-5-methyl-4-Isoxazolepropionic acid (AMPAR), N-methyl-D-aspartic acid (NMDAR), and γ-Aminobutyric acid (GABAR). Given a spike train, (t_*k*_), in the presynaptic neuron *j*, the gating variable *S*, follows the dynamics:

(4) $$\begin{array}{r} \frac{{dS}_{\alpha }}{dt}=\;-{\frac{S_{\alpha }}{\tau }}_{\alpha ,d}\;+x_{\alpha },\frac{dx_{\alpha }}{dt}=\;-\;\frac{x_{\alpha }}{\tau_{\alpha ,r}}\;+\;\underset{k}{\sum }\delta \left(t-t_{k}\right) \end{array}$$

where α denotes G(for GABA), A(for AMPA) and N(for NMDA), respectively. The rising time constants are τ_*G, r*_ = 0.2 ms, τ_*A, r*_ = 0.2 ms, τ_*N, r*_ = 10 ms. The decaying time constants are τ_*G, d*_ = 5 ms, τ_*A, d*_ = 2 ms, τ_*N, d*_ = 100 ms. The postsynaptic neuron i receives synaptic currents *I*_*i, syn*_ = *I*_*i, A*_ + *I*_*i, N*_ + *I*_*i, G*_ with $I_{i,A}=\left(V_{i}-V_{E}\right)\sum_{j}g_{ji,A}S_{j,A},I_{i,N}=\left(V_{i}-V_{E}\right)\sum_{j}\frac{g_{ji,N}S_{j,N}}{1+\left[Mg^{2+}\right]exp\left(-0.062V_{i}/3.57\right)}$, and $I_{i,G}=\left(V_{i}-V_{I}\right)\sum_{j}g_{ji,G}S_{j,G}$, where [Mg^2+^] = 1 mM, *V*_*E*_ = 0 mV, and *V*_*I*_ = −70 mV. We only used the NMDA receptors when we simulated the persistent oscillation in **Figure 9**.

### Background input

The background noise to a network was modeled as uncorrelated Poisson spike trains delivered to each neuron at a rate of *vB* = 1kHz (which can be regarded as the net input from thousands of pre-synaptic neurons). The background noise was exclusively mediated by AMPA receptors (AMPARs) with a maximum conductance of 2.48 nS for pyramidal cells and 1.9 nS for interneurons. In the network with both the E-I and I-I loops, the background inputs to pyramidal cell and interneuron are set to be 1.4 and 1 kHz Poisson spike trains, respectively.

### Numerical method

The second-order Runge-Kutta method was applied to integrate differential equations with a time-step of *dt* = 0.02 ms.

### Measurement of phase shift

The spiking moment of a neuron is taken at the peak of the membrane potential if its value crosses 15 mV, and the size of time bin is *dt* = 1.0 ms. The instant population firing rate *r(t)* is given by the number of spikes in the time window [*t, t*+*dt*] divided by the number of neurons and *dt*. The Fourier transformation was applied to the instant population firing rate to obtain the amplitude and frequency of oscillation. By calculating the cross-correlation function between the instant population firing rates of pyramidal cells and interneurons, we obtained the time lag between two discharges, and the phase shift is given by the product of the oscillation frequency and the time lag.

## Results

### Phase shift in oscillation controlled by the E-I loop

To elucidate the mechanism underlying phase shift clearly, we start to consider a network model whose oscillation is controlled purely by the E-I loop. As shown in Figure 1B, the network consists of two groups, one for pyramidal cells and one for interneurons, and there is no connection between neurons in the same group. Both groups of neurons receive external non-oscillatory excitatory inputs in order to maintain the network activity. It is well-known that such a E-I loop can generate oscillation (Brunel and Wang, 2003; Geisler et al., 2005; Wang, 2010).

We first briefly analyze how the phase shift arises in the E-I loop. In one cycle of oscillation, discharges of pyramidal cells first increase the gating variables of AMPA-mediated synapses (*S*_*A*_), and subsequently the AMPA-mediated currents depolarize interneurons. Consequently, discharges of interneurons increase the gating variables of GABA-mediated synapses (*S*_*G*_), inducing GABA-mediated currents, which feedback and finally suppress the activities of pyramidal cells. In such an interaction cycle, the gating variable *S*_*A*_ lags behind the firing rate of pyramidal cells (*r*_*e*_) by a phase Φ_P, syn_ due to the time consuming of the synaptic dynamics; and similarly the gating variable *S*_*G*_ lags behind the firing rate of interneurons by a phase Φ_I, syn_ (Brunel and Wang, 2003; Geisler et al., 2005). Moreover, the response of a neuron to its input further induces a phase lag, which is denoted as Φ_P, cell_ for pyramidal cells and Φ_I, cell_ for interneurons. The values of Φ_P, cell_ and Φ_I, cell_ depend on the effective membrane time constants of the neurons, and a smaller effective membrane time constant leads to a shorter phase lag (Brunel and Wang, 2003; Geisler et al., 2005). The negative sign of the synaptic currents from interneurons to pyramidal cells contributes a phase lag of 180°. Summarizing all the above phase lags, which forms one cycle, we get Φ_P, cell_ + Φ_P, syn_ + Φ_I, cell_ + Φ_I, syn_ + 180° = 360°. The phase shift from discharges of pyramidal cells (*r*_*e*_) to that of interneurons (*r*_*i*_) equals to Φ_P, syn_ + Φ_I, cell_, which is smaller than 180° according to the above equation, implying that *r*_*e*_ always precedes *r*_*i*_.

**Figure 2:**
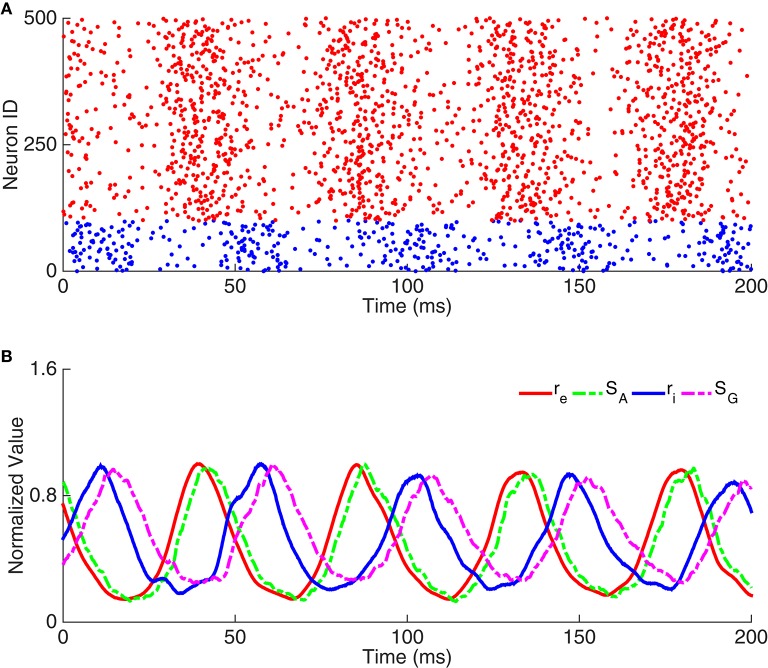
**Example of oscillation exclusively emerging from the E-I loop. (A)** Raster plots of neuron activity. Red dots represent pyramidal cells and blue dots interneurons. Here we only show the activity of 100 interneurons and 400 pyramidal cells. **(B)** Time courses of dynamic variables, including the normalized firing rate of pyramidal cells (r_e_), the gating variable of AMPA receptor (S_A_), the firing rate of interneurons (r_i_), and the gating variable of GABA receptor (S_G_).

We carried out simulation using the network in Figure 1B to validate the above analysis. As shown in Figure 2A, the raster plots of pyramidal cells and interneurons demonstrate that discharges of pyramidal cells indeed precede that of interneurons. Figure 2B displays that the peaks of *S*_*A*_, *r*_*i*_, and *S*_*G*_ fall in the left half side between two peaks of *r*_*e*_, consistent with the property that the phase shift from *r*_*e*_ to *r*_*i*_ is smaller than 180°.

**Figure 3:**
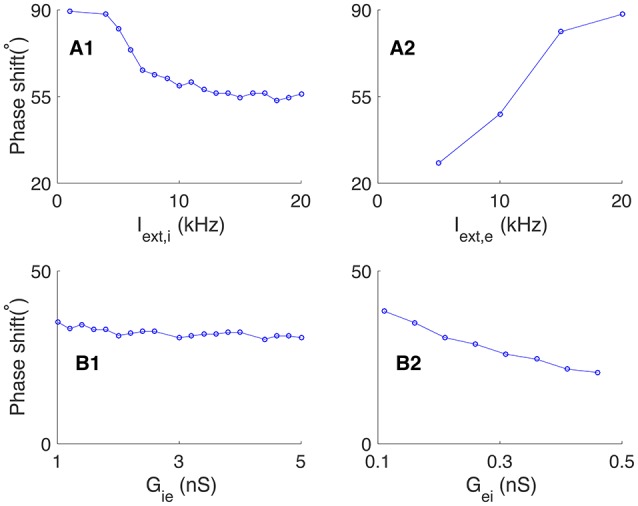
**Effects of external input and connection strength on the phase shift in oscillation generated by the E-I loop alone. (A)** Effects of external input on the phase shift, Φ. **(A1)** The phase shift decreases with the external input to interneurons *I*_*ext, i*_, given fixed *I*_*ext, e*_. **(A2)** The phase shift increases with the external input to pyramidal cells *I*_*ext, e*_, given fixed *I*_*ext, i*_. **(B)** Effects of synaptic conductance on the phase shift, Φ. **(B1)** Phase shift as a function of the synaptic conductance *G*_*ie*_ from interneurons to pyramidal cells, given fixed *G*_*ei*_. **(B2)** Phase shift as a decreasing function of the synaptic conductance *G*_*ei*_ from interneurons to pyramidal cells, given fixed *G*_*ie*_.

We further explored how the phase relationship between neurons is affected by the external excitatory inputs to the network and the synaptic conductance between pyramidal cells and interneurons. We found that: (1) increasing the excitatory inputs to interneurons (*I*_*ext, i*_) alone reduced the phase shift between pyramidal cells and interneurons (see Figure 3A1). This is due to that a stronger excitatory input to an interneuron leads to an effectively shorter membrane time constant, which speeds up the response of interneuron to its input; (2) increasing the excitatory inputs to pyramidal cells (*I*_*ext, e*_) alone enlarged the phase shift (see Figure 3A2). This is due to that a stronger excitatory input to a pyramidal cell leads to an effectively shorter membrane time constant, which makes the pyramidal cell discharge earlier in one cycle; (3) varying the synaptic conductance from interneurons to pyramidal cells has little influence on the phase shift (see Figure 3B1); (4) increasing the synaptic conductance from pyramidal cells to interneurons decreases the membrane time constants of interneurons effectively, which leads to a reduced phase shift (see Figure 3B2).

### Phase shift in oscillation controlled by the I-I loop

We further considered a network model whose oscillation is purely controlled by the I-I loop. As shown in Figure 1C, interneurons are reciprocally connected, and they project to pyramidal cells without feedback. The previous study has shown that characteristic oscillation can arise from the I-I loop via mutual inhibition between interneurons, if the external excitatory input is sufficiently strong (Wang and Buzsáki, 1996). In such a case, the excitatory input depolarizes interneurons to fire, which release neurotransmitters GABA to activate GABA-receptors at the postsynaptic neurons, suppressing the activities of interneurons. Those interneurons will not discharge until the external input activate them again. This process is repeated and the network oscillates.

**Figure 4:**
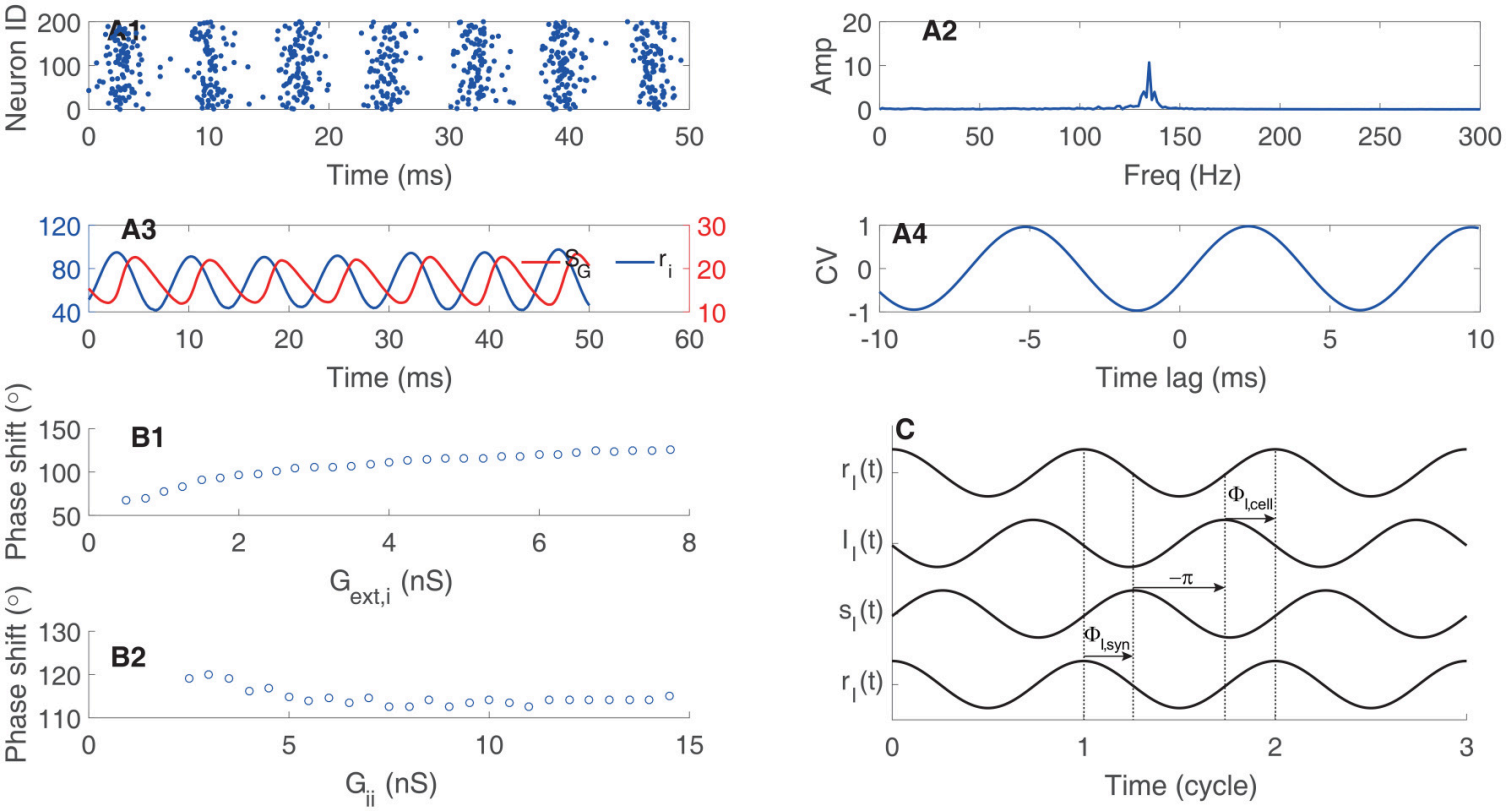
**Phase relationship in oscillation of coupled interneurons. (A)** An example of oscillation in a network with 1000 coupled interneurons. **(A1)** raster plots of activities of 200 interneurons. **(A2)** The amplitude of the oscillation peaks at about 140 Hz. **(A3)** Time courses of the firing rate (*r*_*i*_) and the synaptic variable (*S*_*G*_). **(A4)** The cross-correlation function between *r*_*i*_ and *S*_*G*_. **(B)** The phase shift of *r*_*i*_ with respect to *S*_*G*_. **(B1)** The phase shift is an increasing function of the synaptic conductance of the external input. **(B2)** The phase shift is a decreasing function of the synaptic conductance between interneurons. **(C)** Illustrating the phase relationship between *r*_*i*_ and *S*_*G*_. The response of the gating variable *S*_*G*_ to the firing rate induces a phase lag Φ_I, syn_. The inhibitory synaptic current lags behind the gating variable by 180°. The response of interneurons to the input current induces a phase lag Φ_I, cell_.

We can decompose one circle of oscillation into different components. As shown in Figure 4A, the gating variable of synapse conductance *S*_*G*_ follows the interneurons' firing rate *r*_*i*_ with a phase lag Φ_I, syn_ due to the synaptic dynamics (Brunel and Wang, 2003; Geisler et al., 2005). The response of interneurons to their inputs induces another phase lag Φ_I, cell_ (Figure 4C). Consider that the negative sign of inhibitory currents contribute to a phase lag of 180°, we have Φ_I, cell_ + Φ_I, syn_ + 180° = 360°. Thus, quicker neuronal response leads to a smaller Φ_I, cell_ and a larger Φ_I, syn_; whereas, slower neuronal response leads to a larger Φ_I, cell_ and a smaller Φ_I, syn_. Particularly, the stronger external excitatory input leads to a shorter effective membrane time constant, which means a smaller Φ_I, cell_ and then a larger Φ_I, syn_ (Figure 4B1); whereas, stronger mutual inhibition leads to a longer effective membrane time constant, which implies a larger Φ_I, cell_ and a smaller Φ_I, syn_ (Figure 4B2).

**Figure 5:**
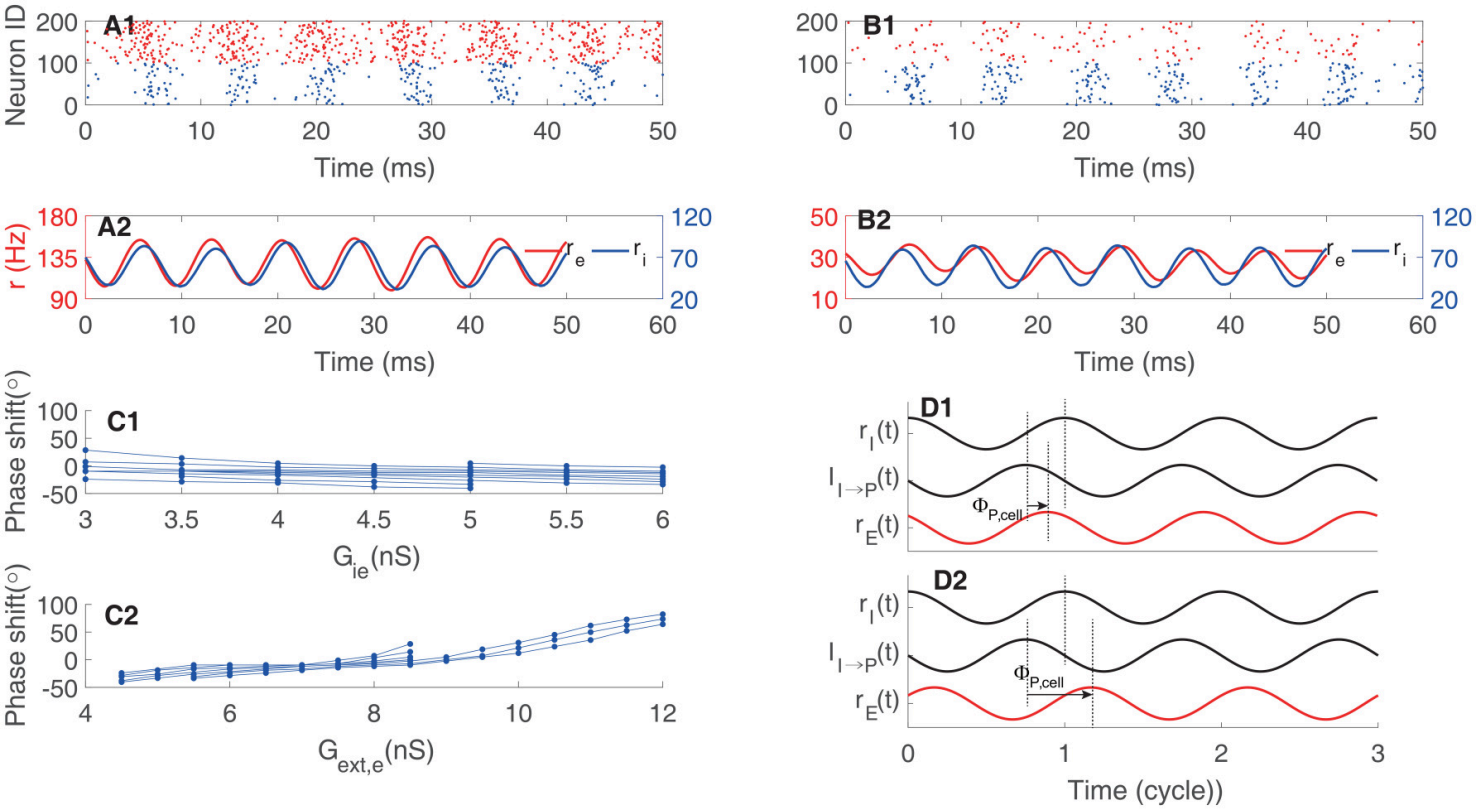
**Phase shift in oscillation determined by the I-I loop. (A)** An example of pyramidal cells preceding interneurons. **(A1)** Raster plot, and **(A2)** firing rate. **(B)** An example of pyramidal cells following interneurons. **(B1)** Raster plot, and **(B2)** firing rate. **(C1)** Phase shift as a function of the synaptic conductance from interneurons to pyramidal cells *G*_*ie*_. One line corresponds to one value of *G*_*ext, e*_. **(C2)** Phase shift as a function of *G*_*ext, e*_. One line corresponds to one value of *G*_*ie*_. **(D)** Illustrating the phase relationship. The phase shift between the synaptic current to pyramidal cells and the firing rate of interneuron is larger than 180° and independent of pyramidal cells. But the phase lag, Φ_P, cell_, induced by the response of pyramidal cell varies. Quick response of pyramidal cells to inputs results in leading phase **(D1)**; whereas, slow response of pyramidal cells to inputs leads to lagging phase **(D2)**. Red for pyramidal cells and black for interneurons.

In a network whose oscillation is controlled by the I-I loop, the oscillatory responses of pyramidal neurons are driven by interneurons (see Figure 1C). We can evaluate the phase shift between pyramidal cells and interneurons accordingly. The gating variable *S*_*G*_ induces a phase lag Φ_I, syn_ with respect to the firing rate *r*_*i*_. The response latency of pyramidal cells to inputs induces a phase lag Φ_P, cell_. Consider that the negative sign of inhibitory currents induces a phase lag of 180°, the total phase shift from *r*_*i*_ to *r*_*e*_ = 180° + Φ_I, syn_ + Φ_p, cell_ (Figure 5D). Therefore, we have: (1) if pyramidal cells have small effective membrane time constants and short response latency, such that the total phase shift is larger than 180° but smaller than 360°, pyramidal cells precede interneurons in oscillation (Figures 5A,D1; note that here we define “leading vs. lagging” based on the amount of phase shift, rather than the actual causal relationship between neuronal responses); (2) if pyramidal cells have large effective membrane time constants and long response latency, such that the total phase shift is larger than 360°, pyramidal cells follow interneurons in oscillation (Figures 5B,D2). Figure 5C2 presents an example that by increasing the external input to pyramidal cells (i.e., larger synaptic conductance *G*_*ext, e*_), which effectively reduces the membrane time constant and the response latency (Geisler et al., 2005), the phase relationship between *r*_*e*_ and *r*_*i*_ can change from lagging to leading. Figure 5C1 presents an opposite example that by increasing the inhibition strength from interneurons to pyramidal cells (i.e., larger *G*_*ie*_), which effectively enlarges the membrane time constant, the phase relationship between *r*_*e*_ and *r*_*i*_ can change from leading to lagging. Overall, depending on the parameters, the phase of pyramidal cells can either precede or lag behind that of interneurons in a network oscillation determined by the I-I loop.

### Phase shift in oscillation determined by competing E-I and I-I loops

In general cases, excitatory and inhibitory neurons in a network are normally reciprocally connected, forming both E-I and I-I loops (Figure 1A), and the network oscillation is a result of the interplay between two loops. Depending on the parameters, the network oscillation may be dominated by either the E-I loop, or the I-I loop, or a mixture of both, and display different phase shift characteristics. We explored how the magnitude of external excitatory inputs (representing modulations from other brain areas) and the strength between excitatory and inhibitory interactions affect the phase relationship between neurons.

**Figure 6:**
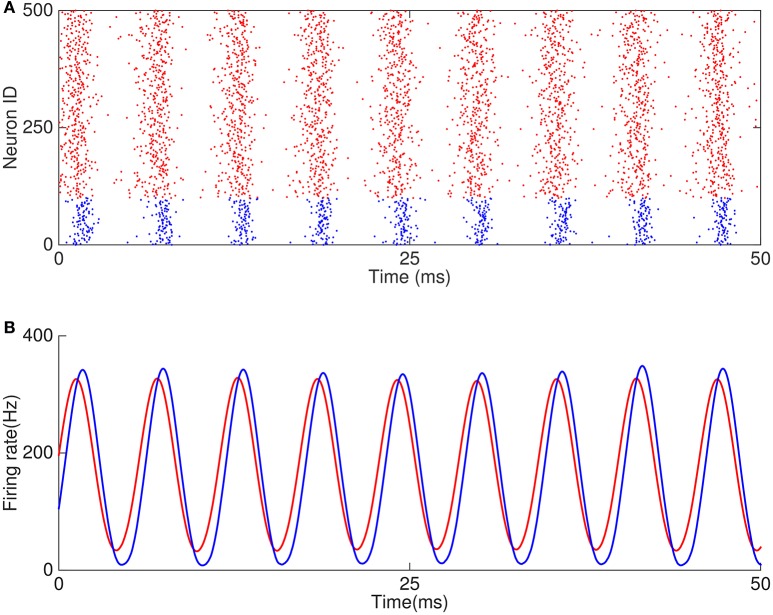
**Example of oscillation caused by E-I and I-I loops. (A)** Raster plots of 500 neurons in the network. **(B)** Firing rates of excitatory and inhibitory neurons.

**Figure 7:**
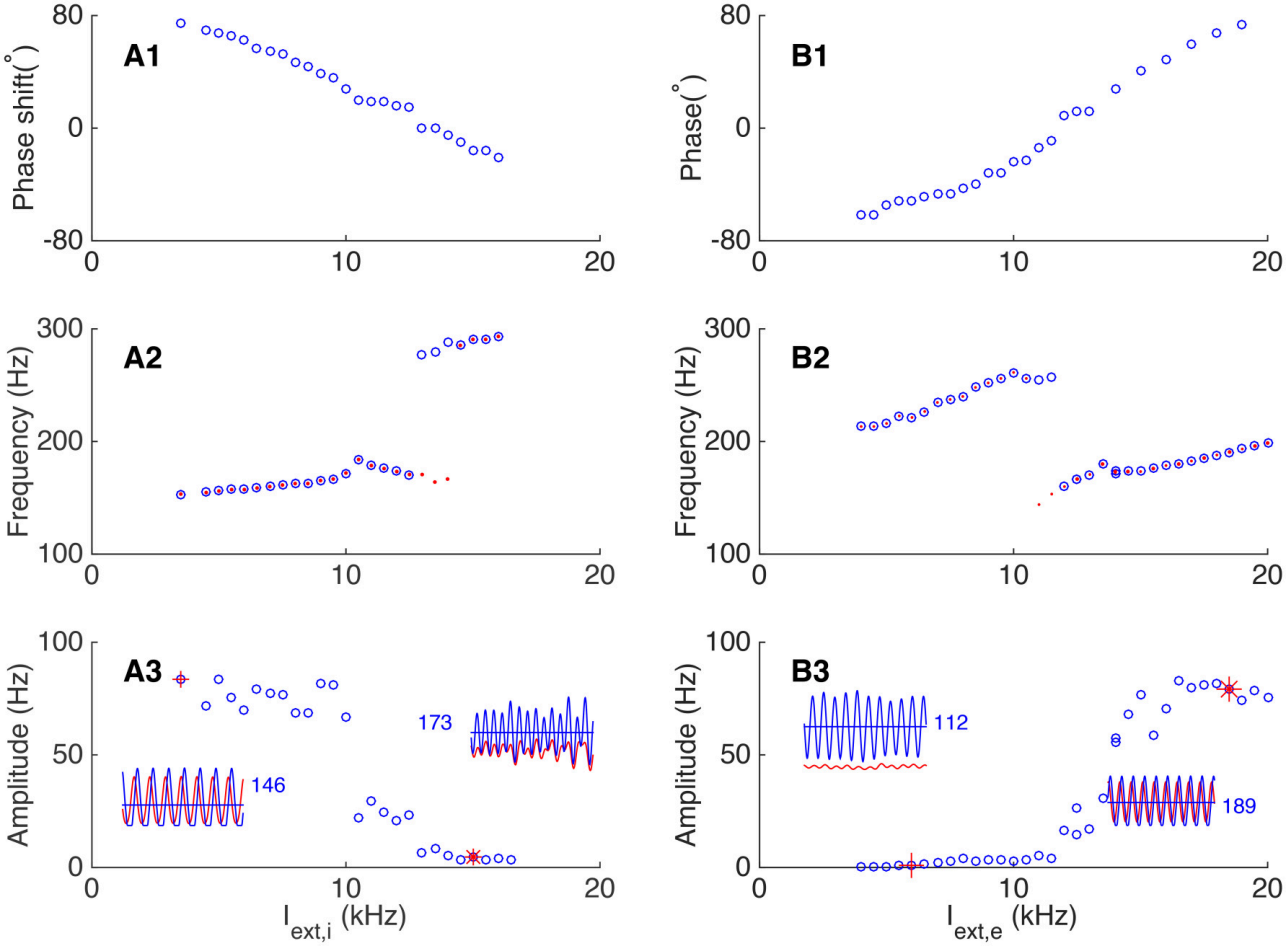
**Dependence of phase shift on external input to the network**. **(A)** Transition from the E-I loop dominating regime to the I-I loop dominating regime occurs with the increase of the external input to interneurons. **(A1)** The phase shift is a decreasing function of the external input to interneurons. **(A2)** The oscillation frequency jumps at the transition point. Red dots (blue circles) denote the frequency of pyramidal cells (interneurons). **(A3)** The oscillation amplitude of pyramidal cells is a decreasing function of the input to interneurons. Left inset shows the firing rates of pyramidal cells (red line) and interneurons (blue line) denoted by red cross, and right inset shows the oscillation of the network denoted by red star. The blue numbers denote the average firing rate of inhibitory neurons. **(B)** Transition from the I-I loop dominating regime to the E-I loop dominating regime occurs with the increase of the external input to pyramidal cells. **(B1)** The phase shift is an increasing function of the external input. **(B2)** The oscillation frequency drops significantly at the transition point. The convention is as same as in **(A2)**. **(B3)** The oscillation amplitude of pyramidal cells is an increasing function of the external input. Convention is as same as in **(A3)**.

We first explored how the external excitatory inputs to interneurons affect the phase relationship between neurons. For comparison, we set a baseline oscillation in which the phase of pyramidal cells leads that of interneurons, as shown in Figure 6. The network parameters fall in the regime where the responses of interneurons are mainly driven by pyramidal cells, and the E-I loop dominates the network oscillation. We then increased the external excitatory inputs to interneurons gradually (*I*_*ext, i*_ from 3.5 to 17 kHz) while kept other parameters invariant, and observed a transition in the network oscillation from E-I dominating to I-I dominating. Figure 7A presents the results, which are: (1) with the increase of the external inputs to interneurons, the phase shift of pyramidal cells with respect to interneurons transits from leading to lagging (from about 80° to about −60°, Figure 7A1). This is because when the excitatory inputs to interneurons become sufficiently strong, the I-I loop dominates the network oscillation. (2) The frequency of the network oscillation increases from about 150 Hz to about 300 Hz with a jump near the transition point between two operating regimes (Figure 7A2). (3) Pyramidal cells and interneurons oscillate at the same frequency when the network oscillation is at a single mode, however, they oscillate at different frequencies near the transition point (Figure 7A2). (4) The oscillation amplitude of pyramidal cells decreases from 50 Hz to few hertz with the increase of the external inputs *I*_*ext, i*_ (Figure 7A3), but the oscillation amplitude of interneurons has little change.

Similarly, we explored how the external excitatory inputs to pyramidal cells affect the phase relationship between neurons by keeping other parameters unchanged. As shown in Figure 7B, starting from a regime where the I-I loop dominates, we increased the external inputs *I*_*ext*,__*e*_ gradually (from 4 to 20 kHz), and observed a transition from I-I loop dominating to E-I loop dominating in the network oscillation. The results are: (1) with the increase of the external inputs, the phase shift of pyramidal cells with respect to interneurons changes from lagging to leading (from about −70° to about 80°, see Figure 7B1). (2) The frequency of the network oscillation increases when either the I-I loop or the E-I loop is dominating, but there is a big drop at the transition point between two oscillating modes (Figure 7B2). Particularly, near the transition point, two or more cycles of interneurons oscillation emerge from the original one cycles of oscillation, while pyramidal cells keep the original cycle. This leads to different oscillation frequency of pyramidal cells and interneurons near the transition point. (3) The oscillation amplitude of pyramidal cells increases from a few Hz in the I-I loop dominating mode to about 80 Hz in the E-I loop dominating mode (Figure 7B3).

**Figure 8:**
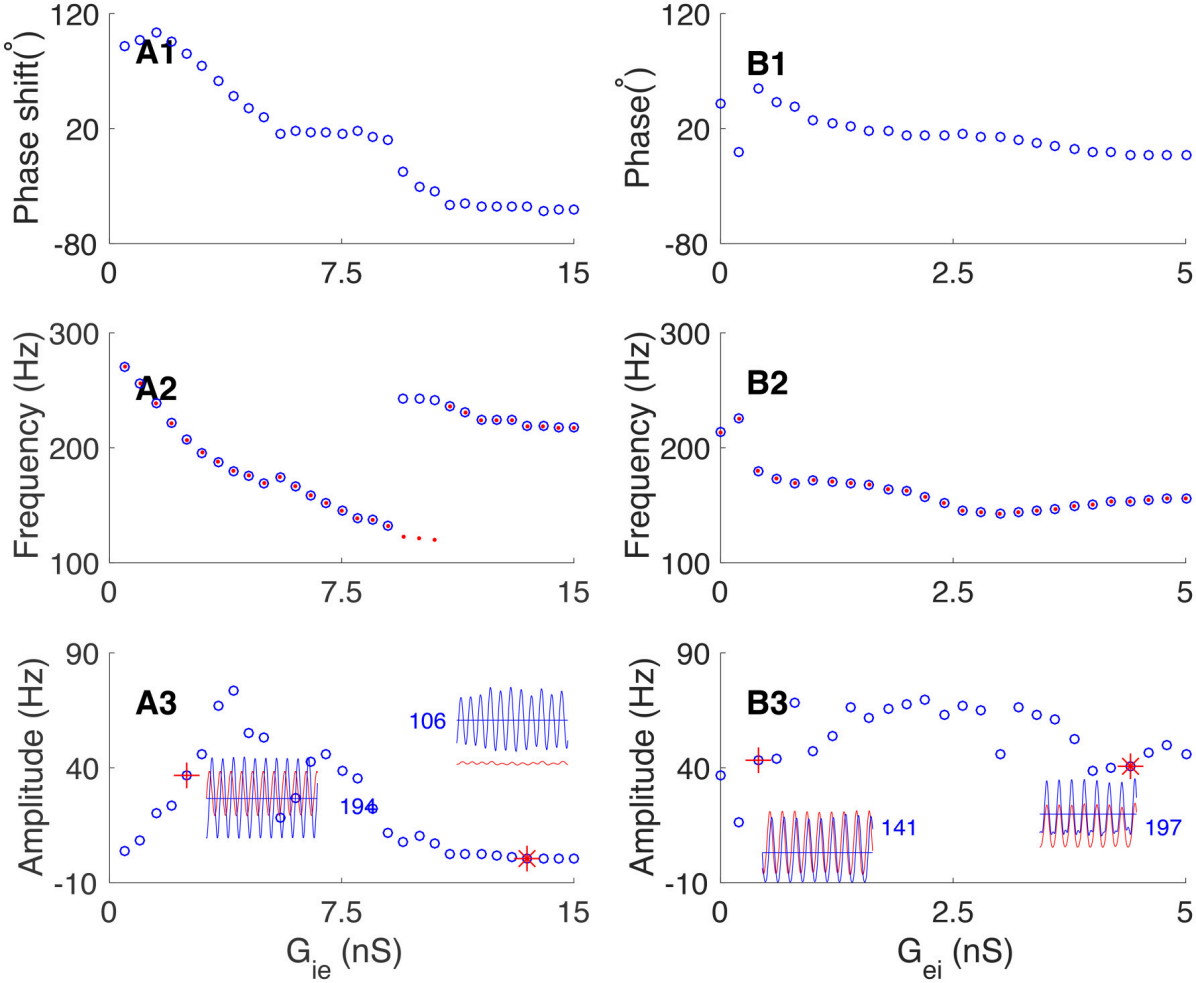
**Dependence of phase shift on the synaptic conductance between pyramidal cells and interneurons. (A)** Transition from the E-I loop dominating regime to the I-I loop dominating regime occurs with the increase of the synaptic conductance from interneurons to pyramidal cells (*G*_*ie*_). **(A1)** The phase shift is a decaying function of *G*_*ie*_. **(A2)** The oscillation frequency is a decaying function of *G*_*ie*_, but it jumps at the transition point. Red dots (blue circles) denote the frequency of pyramidal cells (interneurons). **(A3)** The oscillation amplitude increases at first and then decreases, but the average firing rate of pyramidal cells decreases continuously. Left inset shows the firing rates of pyramidal cells (red line) and interneurons (blue line) denoted by red cross. Right inset shows the firing rates of pyramidal cells and interneurons denoted by red star. **(B)** The network operates in the E-I loop dominating regime with varying *G*_*ei*_. **(B1)** Phase shift as a decreasing function of *G*_*ei*_. **(B2)** Oscillation frequency as a decreasing function of *G*_*ei*_. Convention is as same as in **(A2)**. **(B3)** Oscillation amplitude of pyramidal cells as a function of *G*_*ei*_. Convention is as same as in **(A3)**.

We further explored how the connection strengths between excitatory and inhibitory neurons affect their phase relationship in oscillation. We first increased the inhibition strength from interneurons to pyramidal cells (by increasing the synaptic conductance *G*_*ie*_), and found that the network oscillation can change from E-I loop dominating to I-I loop dominating. Figure 8A presents the results, which are: (1) with the increase of *G*_*ie*_, the oscillating phase of pyramidal cells with respect to interneurons changes from leading to lagging (from 120° to −60°, Figure 8A2); (2) with the increase of *G*_*ie*_, the frequency of network oscillation first decreases gradually from 260 to 120 Hz, and then experiences a sharp upsurge near the transition point when the network oscillation changes from E-I loop to I-I loop dominating (Figure 8A2); (3) The average firing rate of pyramidal cells decreases with the increase of *G*_*ie*_, whereas, the oscillation amplitude of pyramidal cells increases first and decreases subsequently (Figure 8A3).

Similarly, we explored how the connection strength from pyramidal cells to interneurons affects the phase relationship. Increasing the synaptic conductance *G*_*ei*_ has 2-fold effects: on one hand, it increases the excitation to interneurons from pyramidal cells, but on the other hand, it also increases the feedback inhibition to pyramidal cells from more active interneurons. These two effects largely cancel each other. Therefore, we observed that: (1) with the increase of *G*_*ei*_, the phase shift from pyramidal cells to interneurons decreases from about 30° to slightly larger than zero (Figure 8B1). (2) The frequency of network oscillation decreases from about 200 Hz to about 150 Hz (Figure 8B2). (3) The oscillation amplitude of pyramidal cells has only a minor change (Figure 8B3).

### Phase shift in oscillation without external input

**Figure 9:**
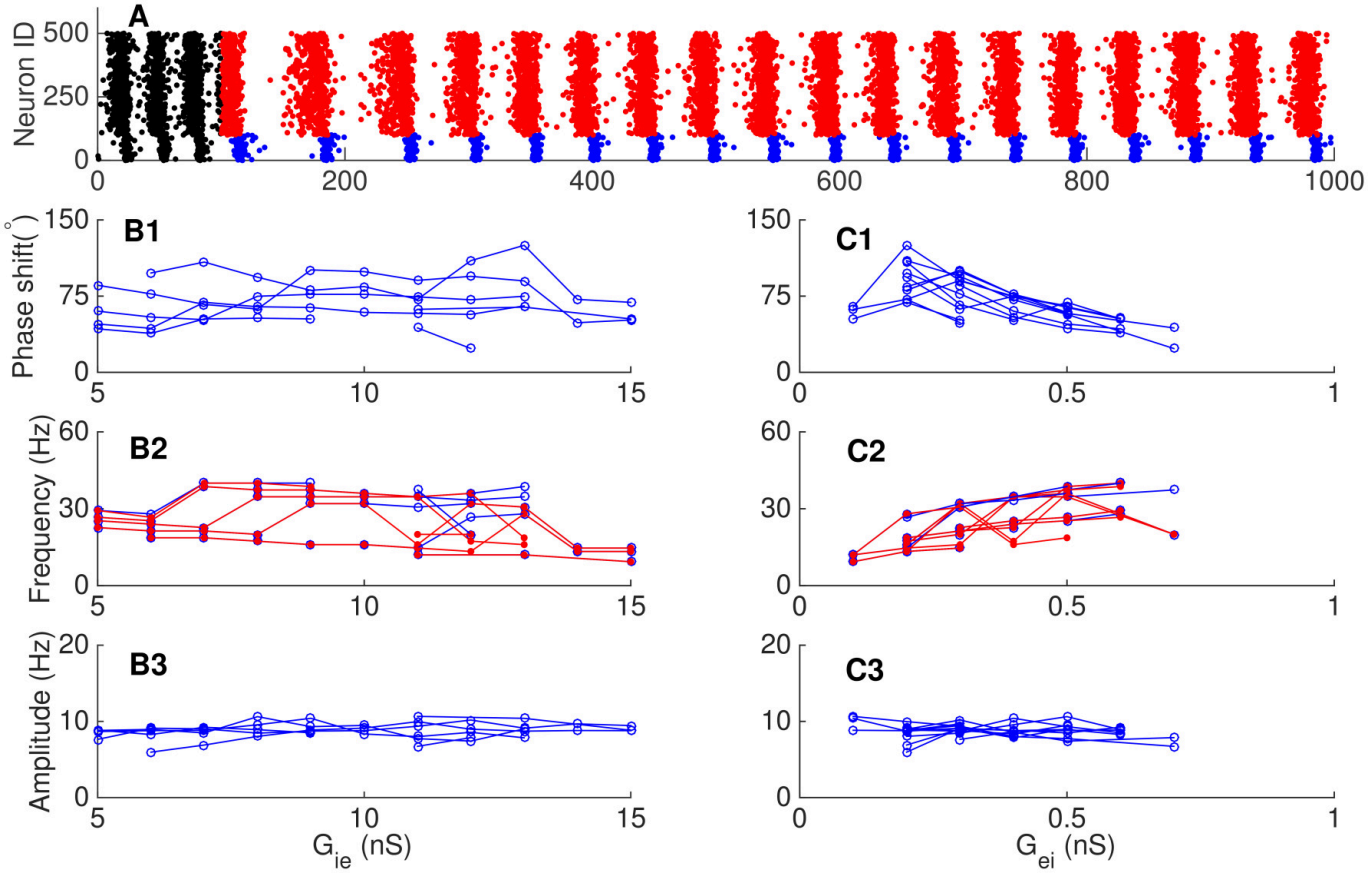
**Phase relationship in persistent oscillation. (A)** Raster plots of persistent oscillation. The external input was withdrawn after 150 ms. Red dots for pyramidal cells and blue dots for interneurons during persistent oscillation. **(B)** Effects of the synaptic conductance from interneurons to pyramidal cells *G*_*ie*_. One line in each panel corresponds to fixed value of *G*_*ei*_. Phase shift **(B1)**, oscillation frequency **(B2)**, and oscillation amplitude **(B3)** as functions of *G*_*ie*_. **(C)** Effects of synaptic the conductance from pyramidal cells to interneurons *G*_*ei*_. One line in each panel corresponds to fixed value of *G*_*ie*_. Phase shift **(C1)**, oscillation frequency **(C2)**, and oscillation amplitude **(C3)** as functions of *G*_*ei*_. Convention is as same as in Figure 8.

In the previous sections, we have considered that the neural networks receive external excitatory inputs and the latter contributes to the network oscillation. It is known that a neural network can also retain oscillation without relying on external drive, but rather depending on the positive feedback interaction between pyramidal neurons via slow NMDA receptors (Tegnér et al., 2002). This persistent activity has been observed in several cortical areas and is proposed to play important roles in higher cognitive functions such as working memory (Fuster and Alexander, 1971; Funahashi et al., 1989) and attention (Ibos et al., 2013). Obviously, in persistent oscillation, the E-I loop always dominates, which drives the I-I loop if the latter exists, and consequently, the phase of pyramidal cells always precedes that of interneurons. We simulated a network consisting of 800 pyramidal cells and 200 interneurons, and included NMDA receptors between pyramidal cells. The results are shown in Figure 9, which are: (1) the network oscillation persists after even the external input is removed; (2) the variation of the synaptic conductance from interneurons to pyramidal cells has little effect on the network oscillation (Figure 9B); (3) the increase of the synaptic conductance from pyramidal cells to interneurons makes interneurons discharge earlier in one cycle (Figure 9C1) and has minor effects on the frequency and amplitude of the oscillation (Figures 9,C2,C3).

## Discussion

Oscillations ubiquitously exist in neural systems, and characteristic phase shifts between different types of neurons during oscillation have been observed in experiments (Fisahn et al., 1998; Csicsvari et al., 2003; Hájos et al., 2004; Hasenstaub et al., 2005; Mann and Paulsen, 2005; Mann et al., 2005a,b; Oren et al., 2006; Gulyás et al., 2010; Vinck et al., 2013; Zemankovics et al., 2013). Previous studies have revealed that either the E-I or the I-I loop formed by neurons can generate oscillation (Fries et al., 2007; Wang, 2010), but none of them has compared the different phase relationships between neurons produced by the two mechanisms. In this study, we considered a network model consisting of both the E-I and I-I loops. In different parameter regimes, the network oscillation can be dominated by either the E-I loop, or the I-I loop, or a mixture of both. We found that pyramidal cells precede interneurons in oscillations dominated by the E-I loop (Freeman, 1968; Wilson and Cowan, 1972; Leung, 1982; Börgers and Kopell, 2005; Orbán et al., 2006); whereas, pyramidal cells can follow or proceed interneurons in oscillations determined by the I-I loop. In analyzing the factors affecting the network oscillation, we found that by either varying the external inputs or the connection strengths between neurons, a transition between different oscillation modes can occur. These results agree with the experimental findings that with stronger inputs, visual cortical neurons in awake monkey discharge earlier in gamma cycle (Vinck et al., 2010), and that injecting currents affects the phase of spiking of a neuron with respect to LFP (Hasenstaub et al., 2016). It is worthy of noting that connections from pyramidal cells to interneurons have different effects on oscillation compared to the reversed connections. This result is different from that in the previous studies (Brunel and Wang, 2003; Börgers and Kopell, 2005; Geisler et al., 2005; Ledoux and Brunel, 2011). Another interesting point is that the oscillation frequency of interneurons is different from that of pyramidal cells near the transition point between E-I loop dominated regime and I-I loop dominated regime, suggesting that pyramidal cells, and interneurons can oscillate at their own frequency. Overall, our study demonstrates that different structures and different parameters of a neural network exhibit different oscillation modes, and they lead to different phase relationships between neurons. This implies that we may use this knowledge to infer the neural circuit property based on the observed characteristic phase shift between neurons. Actually, a recent study found that portion of hippocampal CA1 pyramidal cells have different preferred spike phase with respect to theta or gamma local field potential during wake state and rapid eye movement sleep (Mizuseki et al., 2011). This phase shifting may result from the change of intrinsic properties of neurons and synaptic interaction due to the markedly reduced tonic release of subcortical neuromodulators during REM sleep, including serotonin, norepinephrine, histamine, and dopamine (Pace-Schott and Hobson, 2002). This implies that phase shift between neurons may have cognitive function meanings besides the information relay and spike timing dependent plasticity between neurons (Fries et al., 2007).

In the present study, we explored how externals input and neuronal connection strengths determine phase shift. Other factors, such as those affecting the dynamics of single neurons, may also affect phase shift, and their effects can be analyzed similarly. For instance, there exists different types of subthreshold ionic currents, e.g., the low threshold calcium current (I_T_), the persistent sodium current (I_NaP_), the potassium leak current (I_Kl_), the inwardly rectifying potassium current (I_Kir_), and the fast transient A type potassium current (I_A_), and their contributions on the neuronal dynamics are different. I_T_ and I_NaP_ depolarize pyramidal cells, leading to a shorter effective membrane time constant and quicker response to input; whereas, I_Kl_, I_Kir_ and I_A_ hyperpolarize pyramidal cells, leading to a longer effective time constant and slower response to input. Therefore, if the network operates in the E-I loop dominated regime, I_T_ and I_NaP_ of pyramidal cells tend to enlarge the leading phase of pyramidal cells; whereas, I_Kir_, I_A_ and I_Kl_ of pyramidal cells tend to reduce the leading phase of pyramidal cells.

## Author contributions

All authors listed, have made substantial, direct and intellectual contribution to the work, and approved it for publication.

## Funding

This work was supported by NSFC under grant No. 60974075, 31271169, 31671077, and the Fundamental Research Funds for the Central Universities (DW).

### Conflict of interest statement

The authors declare that the research was conducted in the absence of any commercial or financial relationships that could be construed as a potential conflict of interest. The reviewer YQ and handling Editor declared their shared affiliation, and the handling Editor states that the process nevertheless met the standards of a fair and objective review.
